# Tuning Into the Left Ventricle’s Diastolic Dance Following Pulmonary Valve Replacement—Be Aware!

**DOI:** 10.1016/j.jscai.2024.101346

**Published:** 2024-02-24

**Authors:** Maria Gusseva, Abhay Divekar

**Affiliations:** a Department of Pediatrics, UT Southwestern Medical Center, Dallas, Texas; b Heart Center, Children's Medical Center Dallas, Dallas, Texas

**Keywords:** acute pulmonary edema, left ventricular diastolic dysfunction, transcatheter pulmonary valve replacement

Clinically manifest acute left ventricular (LV) diastolic dysfunction (DD) following transcatheter pulmonary valve replacement (TCPVR) is uncommon, especially in children. We present a case that illustrates the pathophysiology with hemodynamic data and patient-specific biomechanical modeling.

A 9-year-old girl was born with critical aortic stenosis, poor LV function, and endocardial fibroelastosis. LV function normalized following balloon aortic valvuloplasty. She underwent a Ross-Konno procedure (18.0-mm pulmonary homograft) for progressive aortic regurgitation (AR) at 2 years of age. During follow-up, she developed moderate neo-AR, free pulmonary regurgitation (PR), and progressive right ventricular (RV) dilation. Cardiac magnetic resonance imaging showed moderate AR (30% regurgitant fraction), LV ejection fraction of 56%, a dilated RV (end-diastolic volume of 149.5 mL/m^2^ with ejection fraction of 51%), free PR (regurgitation fraction 50%), without RV to pulmonary artery (PA) conduit stenosis. Findings on echocardiography were concordant with those of magnetic resonance imaging; there was evidence of restrictive LV physiology/DD (mitral inflow with abnormal E/A and AR Doppler profile). She underwent uncomplicated TCPVR; at catheterization, the right (mean right atrial pressure, 11 mm Hg) and left-sided filling pressures were elevated (wedge pressure, 18 mm Hg, LV end-diastolic pressure 21 mm Hg); the PA pressure was minimally elevated (32/12, 21 mm Hg). After intervention, there was no RV-PA gradient, but there was dramatic increase in PA pressures (53/31, 40 mm Hg) secondary to significant increase in wedge pressure (30 mm Hg; LV end-diastolic pressure, 33 mm Hg). The patient was treated with aggressive diuresis and did well after procedure with minimal pulmonary edema. She was able to be discharged home without need for escalating respiratory or cardiovascular support.

Utilizing a previously reported biomechanical model, preprocedural and postprocedural hemodynamics were simulated (Figure 1). Patient-specific modeling shows that following TCPVR, there is increase in the effective forward pulmonary flow. In the setting of LVDD, this increased preload results in the LV working on the steeper part of the end-diastolic pressure-volume relationship. Hemodynamics show left atrial hypertension and secondary PA hypertension.

**Figure 1:**
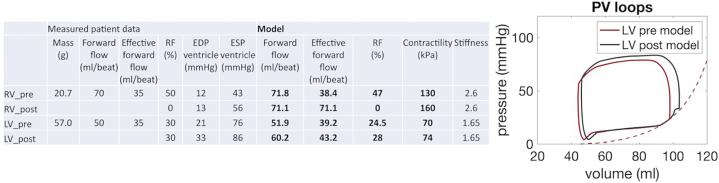
**Biomechanical model.** Biomechanical model showing preprocedural and postprocedural RV and LV numeric data and LV pressure-volume loops. For postprocedural modeling, the passive myocardial properties (stiffness) are kept constant, RV regurgitation fraction is adjusted to 0%, aortic regurgitation is unchanged. Postprocedural volumes are not measured, the preprocedural model is recalibrated to match the postprocedural patient pressure waveforms. Preprocedurally, the LV is working on the steep part of the end-diastolic pressure-volume relationship. Postprocedurally, the LV pressure-volume loop shifts to the right (on the steep part of the end-diastolic pressure-volume relationship) in response to increase in effective forward pulmonary flow. EDP, end-diastolic pressure; ESP, end-systolic pressure; LV, left ventricle; RF, regurgitation fraction; RV, right ventricle; SV, stroke volume.

## Discussion

Lurz et al^1^ and Gusseva et al^2^ have shown that effective RV and LV stroke volumes increase following TCPVR without clinical consequences in the setting of normal LV function. When there is preexisting LVDD, this increase in effective forward pulmonary flow can overwhelm the LV and manifest clinically as acute pulmonary edema, heart failure, and rarely cardiogenic shock.^3^^,^^4^

In addition to severe PR, our patient had moderate AR with LVDD, a finding that is frequent in children with congenital aortic stenosis or mixed stenosis/regurgitation.^5^ Increase in effective forward pulmonary flow following TCPVR increases preload to the LV. When there is preexisting LVDD, the increase in preload results in acute left atrial hypertension and secondary PA hypertension. Clinically, this presents as acute pulmonary edema with or without acute heart failure and cardiogenic shock.

In the setting of acute pulmonary edema or heart failure following TCPVR, coronary compression must be ruled out. Once ruled out, it is important to be aware hemodynamic perturbations discussed. Patients with elevated filling pressures should be treated with diuretics; some may need added support with positive pressure ventilation, afterload reduction (milrinone), and rarely short-term extracorporeal support. Creating an atrial defect may serve to decompress the hypertensive left atrium and allow time for LV adaptation.

Based on an informal survey and published literature LV filling pressures are not always measured following TCPVR, especially in children.^3^ From a practical standpoint, short of universal assessment, LV filling pressures must be assessed in those with known LVDD, those at risk of LVDD (older patients with adult congenital heart disease), and in any patient where there is significant rise in PA pressure following TCPVR.

## Pearls in Hemodynamics from editors Larry S. Dean, MD, and Morton J. Kern, MD

• Following TCPVR, there is increase in effective forward RV and LV stroke volume.
• Increased LV preload can unmask latent LVDD manifesting as acute pulmonary edema.
• Measure LV filling pressures in patients with high baseline wedge pressure, LVDD, and in those who have significant increase in PA pressure following TCPVR.
